# Tight Junction Proteins and Signaling Pathways in Cancer and Inflammation: A Functional Crosstalk

**DOI:** 10.3389/fphys.2018.01942

**Published:** 2019-01-23

**Authors:** Ajaz A. Bhat, Srijayaprakash Uppada, Iman W. Achkar, Sheema Hashem, Santosh K. Yadav, Muralitharan Shanmugakonar, Hamda A. Al-Naemi, Mohammad Haris, Shahab Uddin

**Affiliations:** ^1^Division of Translational Medicine, Research Branch, Sidra Medicine, Doha, Qatar; ^2^Department of Pharmacology and Experimental Neuroscience, University of Nebraska Medical Center, Omaha, NE, United States; ^3^Translational Research Institute, Academic Health System, Hamad Medical Corporation, Doha, Qatar; ^4^Laboratory Animal Research Center, Qatar University, Doha, Qatar; ^5^Department of Biological and Environmental Sciences, Qatar University, Doha, Qatar

**Keywords:** tight junction, claudin, signaling molecules, tumor, metastasis

## Abstract

The ability of epithelial cells to organize through cell–cell adhesion into a functioning epithelium serves the purpose of a tight epithelial protective barrier. Contacts between adjacent cells are made up of tight junctions (TJ), adherens junctions (AJ), and desmosomes with unique cellular functions and a complex molecular composition. These proteins mediate firm mechanical stability, serves as a gatekeeper for the paracellular pathway, and helps in preserving tissue homeostasis. TJ proteins are involved in maintaining cell polarity, in establishing organ-specific apical domains and also in recruiting signaling proteins involved in the regulation of various important cellular functions including proliferation, differentiation, and migration. As a vital component of the epithelial barrier, TJs are under a constant threat from proinflammatory mediators, pathogenic viruses and bacteria, aiding inflammation and the development of disease. Inflammatory bowel disease (IBD) patients reveal loss of TJ barrier function, increased levels of proinflammatory cytokines, and immune dysregulation; yet, the relationship between these events is partly understood. Although TJ barrier defects are inadequate to cause experimental IBD, mucosal immune activation is changed in response to augmented epithelial permeability. Thus, the current studies suggest that altered barrier function may predispose or increase disease progression and therapies targeted to specifically restore the barrier function may provide a substitute or supplement to immunologic-based therapies. This review provides a brief introduction about the TJs, AJs, structure and function of TJ proteins. The link between TJ proteins and key signaling pathways in cell proliferation, transformation, and metastasis is discussed thoroughly. We also discuss the compromised intestinal TJ integrity under inflammatory conditions, and the signaling mechanisms involved that bridge inflammation and cancer.

## Introduction

Epithelial and endothelial cells serve as sentries in most of the living systems by providing protective barriers to the various organs from their surroundings and help maintaining homeostasis (Gibson and Perrimon, 2003; Marchiando et al., 2010a; Cheng and Mruk, 2012). These protective barriers are categorized as tight junctions (TJs), adherens junctions (AJs), and desmosomes. Proteins in the TJ barrier are mainly involved in regulation of intercellular communication and paracellular transport (Abraham et al., 2001). Based on their functions they are classified as anchoring junctions, gap junctions and TJ proteins.

Cell adhesion to the extracellular-matrix is vital for normal cell functioning and proper adhesion is thought to be prerequisite for optimal function of cell surface receptors. Anchoring junction proteins including cadherins, catenins, and integrins are primarily involved in cell surface adhesion (Chattopadhyay et al., 2003). Cadherins are present on the membranes of adjacent cells binding each other at the membranes (Dejana, 2004). Among the catenins, β-catenin, which activates the Wnt signaling pathway (Morin, 1999), is involved in cellular adhesion, growth and differentiation and has been implicated in transition of normal cells to transformed/cancer cells (Zhan et al., 2017). Anchoring or Adherens junctions are responsible for binding with the cytoskeleton thus imparting support as well as signaling hubs, which are important in regulating gene expression (Meng and Takeichi, 2009). Gap junctions help in communication of cells through a set of integral membrane proteins called connexins (Dbouk et al., 2009). Gap junctions help in transport/direct exchange of solutes and molecules between cells. Normal/proper functioning of gap junctions have been shown to play key roles in growth, development and tissue homeostasis (Kandouz and Batist, 2010; Mathias et al., 2010). TJs serve as fortifications for the cell with restricted entry and the only possible transport in a normal functioning healthy cell through a TJ is *via* active transport (Anderson and Van Itallie, 2009). They are also responsible in maintaining/imparting cell polarity. However, with increasing knowledge on TJ biology, both structurally and functionally, their roles have been emphasized to be equally important in cellular signaling cascades with control over growth, development, and differentiation. TJs are formed mainly by occludins, claudins and junctional adhesion molecules (JAM) which will be discussed in more detail in this review (Gonzalez-Mariscal et al., 2003). TJ proteins regulate several key signaling pathways in cancer, also indirectly as interacting partners (Balda and Matter, 2009). Dysregulation of cell junction adhesion has been shown to be heavily implicated in the process of epithelial mesenchymal transition (EMT) (Morris et al., 2008). The dysregulation of these junctional proteins is widely correlated in breast, prostate, ovarian, endometrial, lung, liver and colorectal carcinomas (Martin and Jiang, 2009; Brennan et al., 2010). In addition, the TJ proteins play a major role in maintaining the integrity of the intestinal epithelium and any change like gut inflammation results in the disruption of the intestinal epithelium as seen in inflammatory bowel disease (IBD), such as ulcerative colitis (UC) or Crohn’s disease (CD). The disturbances in TJ epithelial barrier integrity by dysfunctions in intestinal epithelial cell (IEC)–intrinsic molecular circuits that control the homeostasis, renewal, and repair of IECs can also trigger IBD. The present review tries to bring out the connection between various junctional proteins and signaling pathways associated with inflammation and cancer, with major focus on cancer.

## Components of Epithelial Junctions

**Figure 1:**
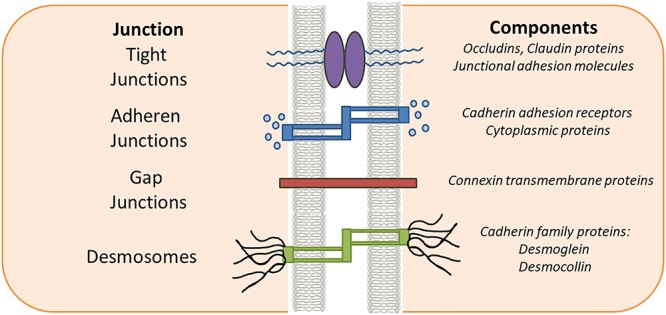
Epithelial intercellular junctions. Schematic drawing of the epithelial junction in vertebrate cell. The tight junctions, adherens junctions and gap junctions are located in the apical most region of the cell while the desmosomes are located toward the basal regions.

The structural integrity and key barrier function of epithelia and endothelia is preserved through interactions involving TJs, AJs, desmosomes and gap junctions (Figure 1). AJs are typically formed between cells and play important roles in development and tissue homeostasis. Desmosomes mainly provide mechanical strength to the cell in conjunction with cytoskeleton. Desmosomes are not continuous and cannot prevent solute transport, instead they create a strong structural network that binds cells together throughout the tissue (Kottke et al., 2006). In contrast, gap junctions are like bridges between two cells allowing passage of nutrients or solute etc. between them. Gap junctions are a family of transmembrane proteins, also called connexins, which play a key regulatory role in cell differentiation and growth. TJs are exclusively found in epithelium and endothelium and are specific to vertebrates. The dysregulation of TJs leads to altered barrier function resulting in changes in levels of inflammatory cytokines such as IFN-α, IFN-gamma, IL-6 and IL-1β as seen in inflammation associated diseases such as IBD, multiple sclerosis and cancer (Harhaj and Antonetti, 2004; Turner, 2006; Cereijido et al., 2007). Therefore, current strategies are being developed by clinicians and researchers to treat these diseases by targeting the compromised TJs. TJs in cancer and inflammation are the main focus of this review.

## Tight Junctions

Tight junctions define the extremes of the cell by demarcating the cells upper and lower regions thus conferring polarity to the cell (Figure 1). Claudins and occludins are the most important TJ proteins that control the vital function of the cells. Other TJ proteins such as cingulin, Pals1 (Proteins Associated with Lin Seven 1), MUPP1 (multi-PDZ domain protein 1), and ZO1, ZO-2, ZO-3 (Zona occludens) (Guillemot et al., 2008) are framework forming proteins connecting transmembrane proteins with the actin cytoskeleton. There are three different ZO-1 proteins with shared structural features, Src homology 3 (SH3) domain, guanylate kinase (GUK) domain and N-terminal region with 3PDZ domains. ZO proteins form the central network for protein interactions. The first PDZ domain of all ZO proteins associates directly with the C-termini of claudins and this association has been attributed to have central role in TJ assembly and function. Down-regulation of ZO proteins has been reported in several cancers such as decreased levels of ZO-1 leads to increased motility in pancreatic cancer (Doi et al., 2012). However, upregulation of ZO-1 expression in melanoma cells has also been reported (Smalley et al., 2005). Abnormal TJs as a result of either inflammation, mutations or an aberrant signaling mechanism disturbs the proper cell functioning and consequently results in disease such as cancer and other abnormalities (Resnick et al., 2005; Runkle and Mu, 2013). Among the TJ proteins, claudins in cancer and inflammation will be the focus of this review.

## Claudins: Backbone of Tight Junction Strands

Claudins are a group of transmembrane proteins which play a critical role, along with other TJ proteins, in the proper functioning of epithelial TJs. Most of the claudins share a common motif, -Y-V in the c-terminal region. There are 27 claudins discovered till date which can be classified as closed, selectively permeable based on their functions (Table 1). The importance of the claudins lies in the fact that the TJs are mainly formed by claudins (Cording et al., 2013). Claudins 1, 3, 5, 11, 14, and 19 belong to closed group and are responsible for water tight stability of the cell. Claudins-10b, and 15 allow passage of cations, and claudins -10a and 17 allow anions while claudin-2 is permeable to both anions and water thus ensuring proper availability of water and ions for cellular functions to be effectively carried out (Gunzel and Fromm, 2012). The claudins along with their elegant interaction with occludins can hold all the proteins of the cytoplasmic milieu (Van Itallie and Anderson, 2004). Owing to the above fact, improper functioning of the claudins have been shown to be responsible to several disease conditions like IBD (Lameris et al., 2013), colorectal cancer (CRC) (Kinugasa et al., 2012), UC (Kinugasa et al., 2010) and in numerous additional cancers, including breast, gastric, pancreatic, prostate, and uterine (Table 1). Similarly, mutations in TJ proteins result in abnormalities as seen in patients with familial hypercholanemia (Anderson and Van Itallie, 2009), ichthyosis, and neonatal sclerosing cholangitis (NISCH) syndrome (Hadj-Rabia et al., 2004). In NISCH syndrome, claudin-1 is lost leading to increased epithelial cell paracellular permeability. Claudins exhibit a variable expression pattern (Table 1) in various cell types and tissues and their expression has been described to be important for membrane functions (Markov et al., 2010), for example, claudin-1 is ubiquitously expressed, while claudin-3 and 4 are restricted to developmental stages and specific cell types (Blanchard et al., 2006; Webb et al., 2013). In the gastrointestinal tract, claudins show a high degree of variability in expression in different segments. In colon cancer, claudin-1 was observed to be having transformative and metastatic potential (Dhawan et al., 2005) while claudin-2 overexpression has been shown to be associated with colon carcinogenesis (Dhawan et al., 2011).

**Table 1:** Dysregulated claudins in various cancers, crosstalk and the outcome.

Tight junction proteins *Claudins 1-20*	Type of cancer	Mechanism of action and signaling molecules involved	Reference
Claudin-1	Human Breast Cancer	Overexpression *via* PDGFRB and cadherin -1 deregulation, resulting in deregulated miRNAs associated with tumor suppression	Majer et al., 2016
		PKC/Claudin-1 signaling pathway involved. Can be controlled *via* inhibiting EMT and its related factors: ZEB1, ZO-1, Slug, Twist, MMP9	Hou et al., 2015
	Human Malignant Glioma Cells	Overexpression of Claudins-1, 2, 3, *via* miR-30A targeting SLUG, suppressing EMT and metastasis	Chang C.W. et al., 2016
		Upregulation *via* miR-203, downregulating SLUG and Vimentin and upregulating ZOI, inhibiting invasion and migration	Chang J.H. et al., 2016
	Hepatocellular carcinoma	Inducing c-Abl-ERK signaling pathway	Suh et al., 2013
	Melanoma	Delocalization to cytosol, increasing MMP-2 and migration	French et al., 2009
	Colon cancer	Notch/Wnt-signaling activation, inhibition of goblet cell differentiation, inducing mucosal inflammation, promoting tumorigenesis	Pope et al., 2014a
	Colorectal cancer	Upregulation of Claudin-1 and occludin *via* phosphorylation of p38 and ERK 1/2	Sun et al., 2015
	Gastric cancer	Overexpressed Claudin-1 associated with β-catenin	Huang et al., 2014
	Squamous cellular carcinoma/Solar Keratosis	Decreased expression and Claudin-2 overexpression resulted in leakier epithelial barrier function consequently damaging skin epithelial resistance	Hintsala et al., 2013
		Overexpression in OSCC patient samples associated with advanced clinical stage and invasiveness	Sappayatosok and Phattarataratip, 2015
	Pancreatic ductal adenocarcinoma	Claudin-1, zinc finger transcription factors, ZEB1/Snail induced expression via eEF-2K mediates cancer cell invasion and metastasis	Ashour et al., 2014
Claudin -2	Breast cancer	Overexpression results breast cancer liver metastasis via promoting cancer cell adhesion to hepatocytes	Tabaries et al., 2012
Claudin-3	Lung adenocarcinoma	EGF-activated MEK/ERK and PI3K-Akt pathways	Zhang et al., 2017
	Breast cancer	Overexpression and delocalization results in tight junction protein deregulation, promoting tumor progression	Todd et al., 2015
Claudin-4	Breast cancer	Overexpression increased cell proliferation/migration, reduces apoptotic rate, regulated by methylation status	Ma et al., 2015
		Claudin-4 associated with tumor aggressiveness and formation of vascular channels	Cui et al., 2015
	Endometrial cancer	Intracellular localization of Claudin-4 involved in signaling to and from the tight junctions	Cuevas et al., 2015
	Gastric cancer	Associated with increased MMP-2 and -9 expression levels, enhancing cancer cell invasion	Hwang et al., 2014
	Nasopharyngeal carcinoma	Overexpression related to advanced stage	Suren et al., 2015
Claudin-5	Glioma	Downregulation associated with increasing permeability and ZO-1, occludin suppression	–
		Downregulation of Claudin-5, ZO-1, occludin mediated by RUNX1 via overexpressed miR-18a, leading to increased permeability	Miao et al., 2015
		Reduced Claudin-5, occludin, and ZO-1 expression via overexpression of miR-181a targeting KLF6, leading to increasing permeability	Ma et al., 2014
		Claudin-5 and occludin downregulation mediated by NOS/NO/ZONAB, leading to enhanced permeability	Liu et al., 2015
Claudin-6	Human adenocarcinoma gastric cancer	Overexpression leads to MMP-2 activation	Torres-Martinez et al., 2017
Claudin-7	Non-small cell lung cancer	Reduced expression leads to metastasis	Kudinov et al., 2016
	Colon cancer	Forced expression in cancer cell lines induces MET, suppresses p-Src and MAPK/ERK1/2 via Rab 25 dependent manner inhibiting tumor growth	Bhat et al., 2015
Claudin-8	Colorectal cancer	Downregulation of Claudin- 8 is associated with tumorigenesis	Grone et al., 2007
	Renal oncocytoma	Claudin-8 and 7 as potential diagnostic biomarkers	Kim et al., 2009; Osunkoya et al., 2009
Claudin-9	Lung cancer	Claudin -9 overexpression is associated with overexpressed MMP-12, supporting tumor cell egression	Sharma et al., 2016
	Pituitary oncocytoma	Overexpression correlated with weak blood vascular endothelium, actin cytoskeleton reorganization, paracellular permeability	Hong et al., 2014
Claudin-10	Lung cancer	Increased expression of Claudin-10 is associated with the development of lung adenocarcinoma mediated by c-fos pathway	Zhang et al., 2013
	Biliary Tract cancer	Decreased expression is observed in intrahepatic bile duct cancer	Nemeth et al., 2009
Claudin-11	Hepatocellular carcinoma	Inhibition via miR-99b targeting 3^′^ UTR of Claudin-11 mRNA is associated with metastasis	Yang et al., 2015
	Cancer-associated fibroblasts (CAF)	Claudin-11 and occludin overexpression is associated with CAF migration via TGF-β secretion	Karagiannis et al., 2014
Claudin 12	Colorectal cancer	Claudin-12 overexpression is associated with the progression	Grone et al., 2007
Claudin-14	Human hepatocellular carcinoma	Low expression observed in patient samples associated with advance stage and downregulated expression results in increased expression and nuclear localization of β-catenin	Li et al., 2016
Claudin-15	Malignant pleural mesothelioma	Overexpressed Claudin-15 serves as potential antiproliferative function	Chaouche-Mazouni et al., 2013
	Colitis cancer	Higher expression observed with colitis cancer	Arimura et al., 2011
	Colon cancer	Claudin-15 overexpression associated with MMP-2 and -9 activation suggesting invasive characteristics	Takehara et al., 2009
Claudin-16	Renal cell carcinoma	Overexpressed Claudin-16 is associated with disrupted barrier function and cell adhesion in cancer cells	Men et al., 2015
Claudin-17	Gastric cancer	Downregulated Claudin-17 is observed in gastric cancer tissue correlated with lymphatic metastasis	Gao et al., 2013
Claudin-18	Lung squamous cell carcinoma	Reduced expression is found in patient samples. Claudin-18 overexpression results in suppression of cell cycle G1/2 phase via p21 increase and Cyclin D1 decrease resulting in inhibition of p-Akt	Akizuki et al., 2017
Claudin-20	Human Breast Cancer	Expression results in reduced TER and no decrease in paracellular permeability. Claudin-20 overexpression displayed aggressive phenotype	Martin et al., 2013

In a recent study in CRC patients, claudin-4 expression loss has been attributed to increased metastasis or enhanced invasiveness of tumors and was found to have a relation with distant metastasis (Suren et al., 2014). Overexpression of claudin-3 and -4 in ovarian cancer cells promotes cancer progression (Agarwal et al., 2005) in both mouse and human ovarian cancer xenografts model (Shang et al., 2012). Further, the role of claudin-4 in pro-angiogenic and enhanced motility in ovarian cancer was also demonstrated (Li et al., 2009). Interestingly, claudins-3 and -4 have been shown to be tumor suppressors as well. Their overexpression decreases Wnt signaling, affects *E*-cadherin expression, and decreases *in vitro* cell migration and invasion. In ovarian cancer, downregulation of claudin-3/-4 promotes tumor growth and metastasis, while less expression of claudin-3/-4 along with claudin-7 results in high malignancy in breast cancer (Prat et al., 2010). On the other hand, high expression of claudin-4 suppresses invasion and metastasis in pancreatic cancer (Michl et al., 2003) while in gastric cancer cells similar inhibition is seen without affecting the cell growth (Kwon et al., 2011). Low expression of claudin-6 supports invasiveness in breast cancer (Osanai et al., 2007), while in gastric cancer cells less expression stimulates invasion, migration, and proliferation (50). Interestingly, claudin-7 functions both as tumor suppressor and promoter. In esophageal squamous cell carcinoma claudin-7 has been shown to enhance cell growth and metastasis (Lioni et al., 2007). In CRC and ovarian cancer, claudin-7 overexpression promotes tumor formation and invasiveness (Johnson et al., 2005; Dahiya et al., 2011). However, in colon cancer, the same claudin-7 was shown to be having tumor suppressor effect (Bhat et al., 2015). Claudin-11, a major component of myelin in central nervous system, is possibly involved in growth and differentiation of oligodendrocytes (Tiwari-Woodruff et al., 2001). In addition, altered expression and localization of several TJ proteins can be detected during inflammation process. First and foremost, claudin-2 abundance increases in various inflammatory diseases, such as CD, UC and celiac disease (Heller et al., 2005; Zeissig et al., 2007). Functionally, this leads to a flux of cations and water via the paracellular pathway into the gut lumen, which gives rise to leak flux diarrhea (92). Also, for claudin-15 an increased expression has been reported in celiac disease (Sandle, 2005). Occludin downregulation has been reported for CD, UC and collagenous colitis (Burgel et al., 2002; Heller et al., 2005; Zeissig et al., 2007). In intestinal cell lines occludin knockdown has been shown to increase macromolecule permeability (Al-Sadi et al., 2011; Buschmann et al., 2013). Based on the above literature, the claudins seem to exist universally from normal tissues, hyperplastic conditions, benign neoplasms, and cancers with differential expression, and their loss or gain of function is linked to inflammation and several malignancies.

## Cross-Talk of Claudins With Signaling Pathways in Cancer

**Figure 2:**
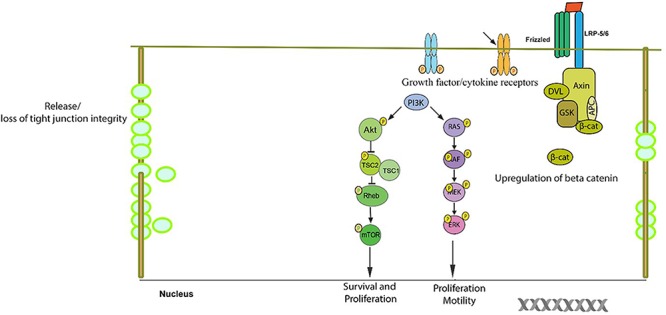
Schematic/proposed signaling model in a cell during tumor formation. Aberrant change in signaling pathways result in the resistance of the normal cellular apoptosis and/or senescence in a cell which is destined to be a tumor cell. The anti-apoptotic proteins belonging to the bcl2 family are upregulated. The tight junction complex changes its course of normal function of selective permeability to unrestricted flow to various unintended solutes/growth/cytokine factors which may be responsible in up regulation of survival signaling pathways. The expression and/or phosphorylation of growth factor/cytokine receptors which promote cell growth are enhanced. The PI3-K/Akt pathway, which is a survival pathway, becomes activated along with the RAS-RAF-ERK pathway and Wnt/beta-catenin pathway which result in the up regulation of several growth response genes.

**Figure 3:**
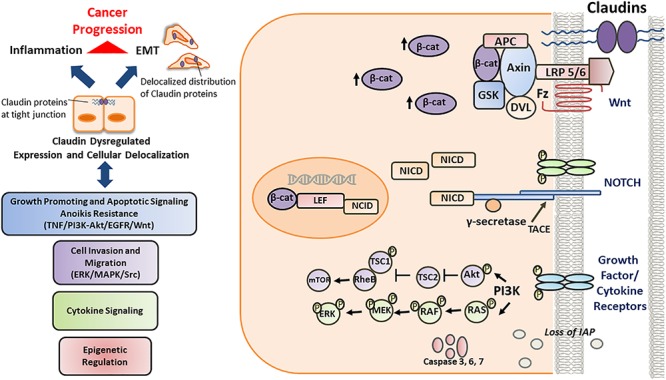
Schematic/proposed signaling model of role and regulation of claudins in a cell during invasive or metastatic stage. Deregulated claudin expression and delocalization occurs as a consequence of epigenetic factors, growth factors and cytokines, inducing loss of “gate and barrier” function and thereby promoting inflammation, EMT and disease progression. Once the cell is destined to be a tumor cell, it further becomes more aggressive. During this stage, the well-regulated junctional molecules between cells become more and more permissible to various factors responsible for the up regulation of the survival, rapid growth and proliferative signaling pathways. Also, the inhibitor for apoptosis (IAP) proteins, which are critical for inhibiting cancer cell death and promoting their survival, are also upregulated. Further, along with the PI3-K/Akt and RAS-RAF-ERK pathways, NOTCH pathway is also upregulated which further enhances the growth potential of the cancer cell. Further, tight junctional protein, such as claudin-1 is associated with beta-catenin and help in the enhanced translocation of beta catenin into the nucleus. At this stage both the NOTCH and the Wnt pathway act in co-ordination to enhance the metastatic potential of the cancer cell.

**Figure 4:**
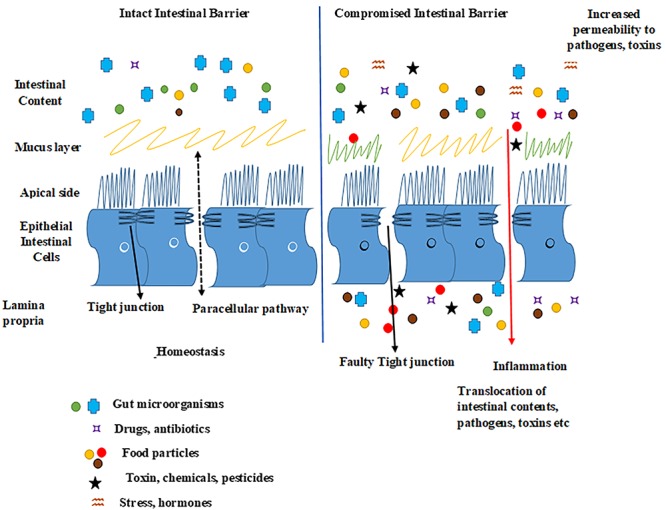
Schematic presentation of healthy and leaky gut. Epithelial tight junctions are intact in a healthy gut and selectively lets some molecules in and out of the intestinal epithelium by functioning as a seal between the neighboring gut cells, hence maintaining homeostasis. Factors such as proinflammatory mediators, microbial gut imbalance, infections, some foods, exposure to chemicals, toxins, or stress may disrupt epithelial tight junctions and increase the intestinal permeability, as well as possibly damage the intestinal barrier by forming tissue lesions and punctures that could lead to a leaky intestinal epithelium. This whole sequence of events may lead to the translocation of undesired luminal gut content (microorganisms, toxins, undigested food particles) into the host tissues activating an immunological response.

Tight junctions of both epithelial and endothelial cells are critical in regulating the permeability across the epithelia and the TJ complex is a hub for signaling pathways which governs the metastatic potential in several cancers. The role of claudins in TJ cancer signaling hasbeen well documented (Figures 2, 3, 4). Mitogen-activated protein kinase (MAPK) (Fujibe et al., 2004) or protein kinase C (PKC) (Nunbhakdi-Craig et al., 2002) induced phosphorylation of claudin-1 and cyclic AMP (cAMP)-induced phosphorylation of claudin-5 (Ishizaki et al., 2003) promotes the barrier function of TJs, while claudin-6 phosphorylation mediated by protein kinase A increases Mg^2+^ transport (Ikari et al., 2008). In addition, claudin phosphorylation is linked to increased paracellular permeability (Yamauchi et al., 2004). Though phosphorylation of claudins is necessary for the maintenance of their function but abnormal phosphorylation affects their aggregation and structural stability which could lead to impaired epithelial barrier function (Sjo et al., 2010; Li et al., 2012). Previously, researchers have demonstrated that during the course of colitis, the phosphorylation status of colonic claudins changes which may be related to the change in the intestinal barrier function and the same group has shown that cytokines play an important role in this process (Li et al., 2015). The phosphorylation of claudins and the associated effects on their normal functions apparently resembles that of the changes in phosphorylation of molecules involved in signaling cascades. This gives us a notion that these two sets of molecules might be closely related in their origins and functions which in due course of evolution might have diversified roles. This would help us to develop common drug targeting strategies.

Very recent studies on blood-brain barrier (BBB) alterations in Japanese encephalitis virus infection (JEV), increases the diverse relationship of TJ proteins and signaling pathways (Wang et al., 2018). It was shown that a decrease in claudin-5, ZO-1 and occludin was observed during JEV infection which were restored with the administration of neutralizing antibodies against IP-10, an abundant chemokine produced in the early stage of JEV infection, helping decrease the BBB damage. This study suggests a very important role for TJ proteins in maintaining BBB (Wang et al., 2018). More importantly, the authors found that the alteration in BBB permeability was due to the nexus between IP-10, TNF- α and c-Jun N-terminal kinase (JNK) pathway, giving another solid proof of cross-talk between TJ proteins, inflammatory cytokines and signaling networks. Another study, establishing the cross-talk between TJ proteins and key signaling pathways was demonstrated by Choksi et al. (2018), through blood vessel epicardial substance (BVES), or POPDC1, a TJ-associated transmembrane protein which has a key role in protecting colonic epithelial integrity (Choksi et al., 2018). BVES modulates epithelial-to-mesenchymal transition (EMT) via junctional signaling pathways (Williams et al., 2011). While investigating its role in colitis, they observed a decrease in claudin-7 and increased ZO-1 protein expressions. While, a significant increase in claudin-2, JAM-A, and Zo-1 mRNA expression was observed. Moreover, they also observed an increase in phosophomyosin light chain 2 (pMLC), which is a key effector in RhoA signaling (Choksi et al., 2018). Their studies demonstrated, several negatively affected TJ proteins with BVES deletion resulting in an increased colonic permeability. More interestingly, previous studies by Reddy et al. (2016) on BVES suggests an enhancing and suppressive effect on Notch and Wnt pathways respectively in BVES -/- mice. An interesting study by Kim et al. (2018) on the exposure of ozone and TJ proteins turned out to be revealing us another tie up between TJ proteins and signaling pathways, the immune signaling networks. They examined primary human lung epithelial cells and mouse models to understand the relationship of TJ proteins in exposure to ozone conditions. In their study, they found that ozone exposure in mice increases TNF- α, IL-4, IL-18, and IL-1b levels along with seemingly concomitant increase in claudin-3, claudin-4ROS, Nrf2, and Keap1 protein expressions and decrease in the lung claudin-14 protein expression. These recent studies take us toward a more better realization of closely knit association between the TJ proteins and signaling circuits and warrants extensive studies on TJ proteins and signaling networks.

In light of the above studies on TJ proteins and signaling pathways in disease condition, leaves us no doubt that TJ proteins significantly affects the cellular processes. It would also be interesting to understand the modulations in TJ proteins in normal development, which is beyond the scope of present review.

## Apoptotic Signaling: Tnf, Pi3K-Akt and Integrin Signaling

Resistance to anoikis results in anchorage-independent growth and EMT which are vital during cancer progression and metastatic colonization (Paoli et al., 2013). Several mechanisms are involved in anoikis resistance of tumor cells of which integrin over-activation of receptors (Haenssen et al., 2010; Deng et al., 2012; Singh et al., 2012) along with appropriate changes in tumor microenvironment significantly contributes toward successful anoikis resistance. It has been observed that Akt, a signaling protein, plays a central role in anoikis resistance by decreasing the proapoptotic proteins, Bad and caspase-9, through its phosphorylation (Jeong et al., 2008) and by upregulating anti-apoptotic protein, Bcl2 expression. Further, in response to integrin-mediated cell attachment, phosphatidylinositol- 3 kinase (PI3K) activates Akt that promotes cell survival (King et al., 1997). Overexpression of claudin-2 in tissue samples from CRC patients was shown to be correlated with cancer progression. A similar trend was also observed in IBD associated CRC tissues. It has been shown that overexpression of claudin-2 increased cell proliferation, anchorage-independent tumor growth in CRC cells *via* EGF receptor (EGFR)-dependent manner (Dhawan et al., 2011). In line with the above studies, Jose et al. group observed an increase in cell migration and anchorage-independent behavior of human colorectal adenocarcinoma (HT-29) cells in association with increased claudin-3 expression mediated by EGF *via* triggering ERK1/2 and PI3K-Akt pathways (de Souza et al., 2013). Singh et al. (2012) have found a novel link between claudin-1 and Src proteins involved in the regulation of anoikis in colon cancer cells through claudin-1/Src/PI3k-Akt/Bcl-2 dependent signaling. This association significantly stimulates the invasiveness and metastasis of colon cancer cells. All these studies support that interactions between claudins and Bcl-2 have a definitive role in tumor metastasis. However, it should not be overlooked that this interaction may be more specific in CRC. Over expression of claudin-1 in MCF-7 breast cancer cell line contributes to anti-apoptotic role under tumor necrosis factor (TNF)-α treatment while the knockdown of claudin-1 increases the susceptibility of MCF-7 cells to TNF-α-induced apoptosis (Liu et al., 2012). The findings by Rabquer et al. (2010) attribute a pro-angiogenic role to JAM-C, while JAM-A was shown to be important in colon inflammation and proliferation of IEC by inhibiting Akt-dependent β-catenin activation (Nava et al., 2011). Both *in vivo* and *in vitro* studies have shown that the loss of JAM-A expression was associated with higher IEC proliferation (Nava et al., 2011). The same group demonstrated that the increased proliferation of IEC involves PI3K and phosphatase and tensin homolog (PTEN)-dependent Akt-mediated β-catenin transcriptional activation. Interestingly an association of loss of JAM-A expression with significantly altered/ or increased expression of claudin-10 and -15 (Kitajiri et al., 2004), results in increased inflammation and paracellular permeability of the IEC. However, occludin or claudin-2 level was not altered in these cells, which hints toward a possible association of trigger to specific claudins.

These studies show how complex links could be made by TJ proteins with cell death pathways, growth and inflammatory responses. This encourages more studies centering TJ proteins with diverse signaling pathways.

## Notch and Wnt Signaling

Notch signaling plays a key role in tumorigenesis either by activating or inhibiting cellular processes such as proliferation, differentiation, and apoptosis (Bolos et al., 2007; Leong and Gao, 2008; Bertrand et al., 2012). In HCT 116 colon cancer cells, Notch represses the p53-dependent transactivation through the interaction of Notch1 with p53 which results in inhibition of p53 phosphorylation, and subsequent inactivation of p53-dependent apoptotic pathway (Kim et al., 2007). Notch and Wnt/β-catenin signaling is important for intestinal development and maintaining homeostasis (Ahmed et al., 2012). The Notch signaling is also important in determining intestinal epithelial renewal and their function (Fre et al., 2005). On the other hand, Wnt signaling pathway by regulating the cytoplasmic and nuclear β-catenin levels plays a crucial role during development of different tissues and organisms (Clevers and Nusse, 2012). Few reports have shown upregulated expression of Wnt target genes, c-myc, cyclin-D1, MMP-7, Tcf1, and EphB2, and Notch target gene hes1 in tumors (van de Wetering et al., 2002; Rodilla et al., 2009). Moreover, lack of coordination between Notch and Wnt signaling was shown to be involved in enhancing inflammation or tumorigenesis (Fre et al., 2009; Ahmed et al., 2012). In CRC cell lines, claudin-1 expression enhances the tumorigenic ability and also leads to the mucosal inflammation *via* activation of Notch pathway, and further inhibits goblet cell differentiation (Pope et al., 2014a). It has been observed that caudal-related homeobox (Cdx) transcription factors regulate claudin-1 gene expression in human colon cancer cells and functional crosstalk with Wnt-signaling pathway was found to be important for this regulation (Bhat et al., 2012). In accordance with these studies, Notch-signaling was shown to be regulated by claudin-1 overexpression, which in turn increase the MMP-9 and p-ERK expression in transgenic mice resulting in metastasis of colon cancer and colonic epithelial homeostasis (Pope et al., 2014b). Added to the growing complexity, it has been demonstrated recently that claudin-7 to be a tumor promoter, in colon and pancreatic cancer, through its association with epithelial cell adhesion/activating molecule (EpCAM) thereby promoting/inducing EMT (Philip et al., 2014). By disrupting the link between β-catenin and F-actin, EpCAM interferes with *E*-cadherin mediated cell-cell adhesion (Thuma and Zoller, 2013). It also has a role in Wnt/β-catenin signaling pathway (Yamashita et al., 2007; Lin et al., 2012), regulates PKC (Maghzal et al., 2010) and MMP-7 expression as well (Denzel et al., 2012). It was shown that claudin-7 guides/recruits EpCAM toward signal transduction platforms or glycolipid-enriched membrane microdomains (GEM) where it becomes susceptible to digestion by TNF-α converting enzyme (TACE) releasing EpIC which acts as a cotranscription factor in cooperation with β-catenin and others (Philip et al., 2014). In addition, EpIC also contributes to EMT by upregulating vimentin, Snail, Slug and downregulating *E*-cadherin. Interestingly, Notch was also upregulated in holoclones, a colony-forming stem cells that have higher growth potential due to absence of differentiated cells (Eglen et al., 1989). Moreover, FGF and TGFβ, known to upregulate EMT (Shirakihara et al., 2011) were down-regulated in claudin-7 knockdown cells.

Activation of Wnt/β-catenin signaling pathway by Wnt ligands is involved in regulating embryonic development and homeostasis in later stages (Lickert et al., 2000; Davidson et al., 2012). Mislocalization of β-catenin and dysregulation of Wnt/β-catenin signaling pathway is shown to be associated with development of various cancers (Polakis, 2012; Keerthivasan et al., 2014; Wang et al., 2014). In CRC, Wnt/β-catenin signaling becomes more important as greater than or nearly 70% of CRC tumors exhibit mutations in *adenomatous polyposis coli (APC)*, a Wnt pathway component. Interestingly nuclear localization of claudin-1, along with β-catenin, was observed in liver metastatic lesion samples (Dhawan et al., 2005) suggesting that claudin-1 may assist/promote the translocation of membranous β-catenin to enhance the activation of its target genes leading to robust growth and/or survival of the cancerous cells. These different important interactions of the TJ proteins with the signaling cascades suggests that TJ proteins might be having different binding specificities to different signaling molecules and that they are dependent in a contextual manner, which needs to be explored.

These studies establish an association of TJ proteins with well established growth and developmental pathways. However, it would be interesting to know about novel signaling mechanisms which may work independently or in association with established pathways keeping TJ proteins in the focus. These studies would open avenues for new strategies of treatment.

## Kinase Signaling

It has been observed that manipulating claudin-1 expression results in phenotypic changes significantly effecting growth and metastasis of tumor xenograft in athymic mice (Dhawan et al., 2005). The same group observed that upregulation of claudin-1 enhanced the metastatic potential by altering the *E*-cadherin expression and Wnt/β-catenin signaling (Dhawan et al., 2005). Interestingly, increased claudin-1 expression in metastatic tissues was associated with its mislocalization from membrane to nucleus (Dhawan et al., 2005). Given the cross-talk between Wnt/β-catenin signaling and NF-κB in inducing inflammatory responses (Ma and Hottiger, 2016), it is possible that claudin-1 associated modulation in signaling may also result in inflammation associated changes. This would be an interesting area to explore in future. Also, in oral squamous cancer, claudin-1 upregulates MMP activity and promotes invasiveness (Dos Reis et al., 2008). In melanoma as well, similar correlation was reported (Leotlela et al., 2007). In human liver cells, increased expression of claudin-1 both at mRNA and protein levels associated with PKC activation, which subsequently promotes invasiveness through stimulation of c-Abl-PKC signaling (Yoon et al., 2010; Lin et al., 2013). Lin et al. have showed that absence of claudin-3 and claudin-4 enhanced the EMT activity in ovarian cancer cells through downregulating *E*-cadherin expression, upregulating Twist, and activating the PI3K pathway (Lin et al., 2013). As PI3K pathway is well evidenced to have roles in recruiting inflammatory immune cells, it may be plausible that claudin modulation has an indirect effect on inflammation as well (Hawkins and Stephens, 2015). Claudin-1 promotes EMT in human liver cells, while claudin-3 and claudin-4 promote EMT in ovarian cancer cells, which suggests that the effect of claudins on EMT is tissue-specific (Yoon et al., 2010). Phosphorylation is shown to be having a regulatory role in the function of claudin-3 and claudin-4, for example, activated PKA (D’Souza et al., 2005) or PKC (D’Souza et al., 2007) phosphorylates claudin-3 and claudin-4 and enhance the paracellular permeability in ovarian cancer cells through the mislocalization of claudins. In human pancreatic cancer cells, phosphorylated claudin-4 by PKC not only increase its mislocalization but also compromised the TJ barrier integrity (Kyuno et al., 2011). Studies have shown that the effects of claudin-3 and claudin-4 are more pronounced in ovarian cancer cells. The overexpression of claudin-3/-4 correlates to ovarian cancer progression with concomitant activation of MMP resulting in increased invasiveness (Agarwal et al., 2005). It was shown that claudin-3 inhibition with small interfering RNA reduced the growth and metastasis of ovarian cancer in xenografts model, which strongly supports the cancer-promoting role of claudin-3 (Huang et al., 2009). Both *in vitro* and *in vivo* studies in ovarian cancer observed that claudin-4 promotes the angiogenesis by inducing the production of angiogenic factors such as IL-8 (Li et al., 2009), suggesting the pro-angiogenic role of claudin-4 in ovarian cancer. On the other hand, adherens were shown to be responsible in the altered expression of claudin-5. In this study, Andrea et al. showed the up regulation of claudin-5 gene by endothelial VE-cadherin (VEC), which transfers intracellular signals at AJs (Taddei et al., 2008). This was achieved by inhibiting the β-catenin translocation to the nucleus or sequestering it from the nucleus and through Akt mediated inactivation of FOXO1 inhibitory activity (Taddei et al., 2008). The treatment of the VEC-positive cells with glycogen synthase kinase 3 (GSK-3) β downregulated the claudin-5 expression. The β-catenin was also found to be directly associated with FOXO1 and that this association at the promoter region of claudin-5 is required for its regulation/overexpression (Taddei et al., 2008). In the absence of VEC, the FOXO1–β-catenin–Tcf-4 complex binds to the promoter of the claudin-5 gene and inhibits its expression. In the light of these studies, it is evident that β-catenin pathway plays a central role in effecting signaling cascades and it also seems to be imperative that it might have influence in inflammation associated mechanism. Another interesting study observed that the co-localization of claudin-9 and -6 with AJs regulatory proteins in a heterologous system forms a novel TJ strand (Nunes et al., 2006). However, this was carried out in normal inner ear cells; it would be interesting to investigate the existence of similar kind of associations in cancer cells and their relevance to cancer metastasis.

## Erk Pathway

ERK signaling is activated by diverse mechanisms which majorly includes ligation of receptor tyrosine kinases and cell adhesion receptors. Activated ERK can phosphorylate a wide range of substrates and thereby affecting a broad array of cellular functions including proliferation, survival, apoptosis, motility, transcription, metabolism and differentiation. In a recent study it was shown that MAPK/ERK1/2 pathway is involved in the regulation of TJ proteins in the mouse epididymis. The study reported that the reduction in ERK1/2 phosphorylation (pERK), is associated with the decrease in ZO-2 expression and increase in ZO-3 expression in TJs but had no effect on ZO-1 expression. In addition, it was shown to affect the redistribution of claudin-1 and claudin-4 at the membrane junctions without affecting claudin-3 (Kim and Breton, 2016). The contradictory role of ERK activation is more pronounced in TJ integrity where its activation leads to disruption of TJs in some epithelial monolayers and prevention in other epithelia. This interesting phenomenon was observed in Caco-2 cell monolayers by Aggarwal et al. (2011). They observed that in under-differentiated Caco-2 cells, ERK is involved in the destabilization of TJs, whereas a protective role was observed in differentiated cells. They suggested that this differential effect is due the differences in the subcellular distribution of ERK and its ability to regulate the association of PKCζ and PP2A (protein phosphatase 2A) with TJ proteins (Aggarwal et al., 2011). ERK signaling has also been shown to be activated by TJ proteins which in turn determines the fate of cell. Though claudin-7 contributes toward cell growth and metastasis of esophageal squamous cell carcinoma (Lioni et al., 2007), in lung cancer it inhibits migration and invasion *via* ERK/MAPK signaling pathway. ERK/MAPK signaling pathway inhibited by claudin-7 caused reduced migration and invasion ability of non-small lung carcinoma cells (Lu et al., 2011). Interestingly, they observed stable complex formation, in co-immunoprecipitation studies, between claudin-7 and claudin-1 and -3 suggesting a cooperative relationship between claudins. Similar results were observed in CRC cells where overexpression of claudin-7 inhibited proliferation and invasion by regulating ERK and Src signaling (Bhat et al., 2015). Another study by Suh et al. (2013) showed that claudin-1 induces EMT in human liver cells, which largely depends on the activation of the c-Abl-Ras-Raf-1/ERK1/2 signaling pathway. This finding supports the importance of c-Abl-ERK signaling in claudin-1 associated malignant phenotype. In lung cancer A549 cell line, the increased expression of claudin-2 was associated with the activation of EGFR/MEK/ERK signaling pathway (Ikari et al., 2012). It was further observed that c-Fos, a down-stream target in an EGFR/MEK/ERK pathway, upregulates the transcriptional activity of claudin-2 by interacting with the AP-1 binding site of claudin-2 promoter (Ikari et al., 2012). Contradictory to ERK activation by claudin-2, Lipschutz et al. (2005) demonstrated that the ERK 1/2 signaling pathway is a negative regulator of claudin-2 expression in mammalian renal epithelial cells affecting TJ permeability and renal epithelial function. These studies give us an indication that the claudins are regulated in a tissue specific manner and they themselves regulate the signaling pathways in the same fashion.

In addition, TJ protein expression and localization changes during inflammation process are well reported. First and foremost, claudin-2 abundance increases in various inflammatory diseases, such as CD, UC and celiac disease (90, 89, 91). Functionally, this leads to a flux of cations and water via the paracellular pathway into the gut lumen, which gives rise to leak flux diarrhea (92). Also, for claudin-15 an increased expression has been reported in celiac disease (91). Occludin downregulation has been reported for CD, UC and collagenous colitis (90, 89, 96). In intestinal cell lines occludin knockdown has been shown to increase macromolecule permeability (98, 99). Based on the above literature, the claudins seem to exist universally from normal tissues, hyperplastic conditions, benign neoplasms, and cancers with differential expression, and their loss or gain of function is linked to inflammation and several malignancies.

In view of the importance of kinase signaling cascades in inflammation and cancer, and the above observed important associations of claudins and kinase pathways, it greatly widens the diverse roles of TJ proteins in smooth functioning of cellular processes. More studies are warranted to delve into the details of cross-talk between TJ proteins and signaling mechanisms not only in cancer but also in other diseases.

## Tight Junction in Intestinal Inflammation and Functional Crosstalk With Signaling Pathways

**Figure 5:**
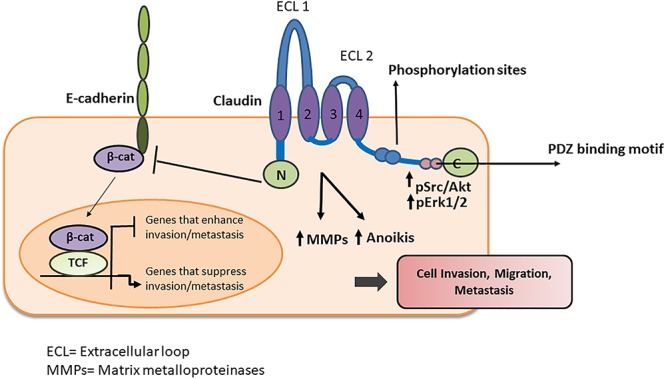
Schematic representation of claudin interaction with adhesion molecules and signaling proteins. Claudins and claudin containing complexes influence diverse signaling processes within cancer cells that results in altered migration, invasion and metastasis. Claudins either interact directly with other adhesion molecules or recruit signaling proteins to execute their diverse array of functions.

As TJ barrier dysfunction and inflammation are tightly associated with each other, equally inflammation and cancer are closely linked (Coussens and Werb, 2002; Raposo et al., 2015; Korniluk et al., 2017). Whether barrier dysfunction is the underlying cause of inflammation or vice versa and if inflammation leads to cancer or vice versa, these are the concepts which need more visibility and discussion. From the literature, it seems that there exists a positive feedback loop which connects them together. In this section we will focus on TJ barrier dysfunction and inflammation and in the next section we will briefly describe the bridge between inflammation and cancer. It is well established that the dysfunction of TJ barrier under inflammatory conditions contributes to the pathogenesis of intestinal disease. Compromised TJ barrier increases paracellular permeability and triggers an array of events including apoptosis, erosion, and ulceration that contributes to intestinal epithelial damage (Zeissig et al., 2004; Heller et al., 2005; Schulzke et al., 2006). Influx of immune cells into the intestinal mucosa *via* disrupted TJ influences the epithelial function by stimulating the release of proinflammatory cytokines such as TNF-α and IFN-γ. Increased levels of TNF-α and IFN-γ in the mucosa of patients with IBD, contributes to the proinflammatory cascade, and in turn intestinal barrier disruption (Madara and Stafford, 1989; Adams et al., 1993; Schmitz et al., 1999; Bruewer et al., 2003) (Figure 5). TJ in inflamed epithelia of the intestine is characterized by reduced TJ strands, strand breaks, and changes in TJ proteins composition and function. Mucosal inflammation affects the permeability of the gut barrier by altering the intestinal epithelial homeostasis that may impair the structure and remodeling of apical junctions. It is now clear that IBD can be triggered by disturbances in TJ barrier integrity via disturbances in IEC molecular machinery that controls the homeostasis, renewal, and repair of IECs. Although TJs are considered a part of the physical barrier, specialized IECs (IECs), such as goblet cells and Paneth cells, play an important role in antimicrobial defense, thus making them crucial to innate immune system. Goblet cells help to protect against invasive pathogens by secreting antimicrobial molecules, such as trefoil factors and mucins. Trefoil factors help in restoring the gastrointestinal mucosal homeostasis while mucin constitutes a thick mucus layer to prevent excessive direct contact of bacteria to the epithelial cell surface (McCauley and Guasch, 2015; Aihara et al., 2017). Paneth cells are involved in the innate host defense by secreting high levels of antimicrobial peptides within the crypts of the small intestine (Ayabe et al., 2004; Kopp et al., 2015). The induction of these antimicrobial peptides is profoundly related with the function of intestinal barriers and hence an association with the IBD (Kim, 2014). Previously, we have shown that claudin-1, most widely studied member of TJ protein family, helps regulate the intestinal epithelial homeostasis by regulating the Notch signaling (Pope et al., 2014b). Increased claudin-1 expression activates Notch-signaling through stimulation of MMP-9 and p-ERK signaling pathway and the overall effect is inhibition of goblet cell differentiation (Pope et al., 2014b). Active inflammatory areas have been shown to possess increased expression of claudin-1 which further contributes to disease severity (Weber et al., 2008). Claudin-3, -5, and -8, function as sealing TJ proteins, whose expression was diminished in patients with CD resulting in impaired TJ complexity, lower number of TJ strands and more strand breaks. These patients also have diminished levels of occludin and upregulated level of pore forming claudin-2 expressed in the ileum of both quiescent and active CD. In addition, colonic biopsies from CD patients showed the mislocalization of claudin-2 contributing to the disrupted TJs. However, other studies have reported increased claudin-12 not claudin-2 expression in ileum of CD patients, the contradictory decreased claudin -2 expression in the sigmoid colon (Lameris et al., 2013). Not only the expression but also the distribution of TJ proteins is affected in inflamed intestinal mucosa as observed with claudin-5 and -8 in the TJ of CD (Zeissig et al., 2007). In case of UC, similar changes in TJ proteins were observed including decreased expression of occludin, claudin-1 and claudin-4 and up-regulation of the pore-forming claudin-2 (Heller et al., 2005). Increased claudin-2 expression both at protein and transcriptional levels was found to be correlated with disease severity in UC (Heller et al., 2005). Additionally, extrajunctional mislocalization of claudin -4 and reduced staining intensity on surface epithelium for claudins- 3, 4, and 7 has been shown in UC (Prasad et al., 2005; Oshima et al., 2008). Additional TJ proteins that were upregulated in UC includes claudin-12 and claudin- 18, however, the elevated claudin -18 expression was not associated with the severity of inflammation indicating a primary defect in barrier function (Zwiers et al., 2008; Lameris et al., 2013).

Increased or abnormal expression of proinflammatory cytokines contributes to the barrier defects in IBD. Patients with IBD, such as UC and CD, are at increased risk of developing CRC, confirming that chronic inflammation predisposes to development of tumors. CRC therefore represents a paradigm for the link between inflammation and cancer. Inflammation is driven by soluble factors, cytokines and chemokines, which can be produced by tumor cells themselves or, more often, by the cells recruited to the tumor microenvironment. Inflammatory cytokines and chemokines promote growth of tumor cells, perturb their differentiation, and support the survival of cancer cells. In CD, the levels of TNF-α and IFN-γ are increased favoring Th1 profile while the inflammatory response in UC is attributed to increased levels of TNF-α and IL-13. Cell culture and animal studies have clearly shown that these proinflammatory cytokines induce changes in TJ proteins, induction of apoptosis and enhanced bacterial translocation as observed both in CD and UC (John et al., 2011). Cytokines affect TJs by regulating the expression and redistribution pattern of proteins. Claudin-2 protein expression was found to be increased in HT-29/B6 cells when treated with TNF-α and IL-13 via the phosphatidylinositol-3-kinase pathway (Mankertz et al., 2009). Native rat colon when exposed to TNF-α and IFN-γ showed increased expression of pore forming claudin-2 and down regulation of barrier forming claudin-1, -5 and -7 (Amasheh et al., 2009). Colonic epithelial cells exposed to TNF-α have shown redistribution of ZO-1 from the cell membrane along with increased paracellular permeability and decreased TER (Schmitz et al., 1999). The changes in TJ structure and the expression of its component proteins on exposure to TNF-α are mediated *via* NF-kB signaling (Soler et al., 1999; Ma et al., 2004). Both human IBD and experimental models of intestinal inflammation showed similar structural and functional changes in TJ (Schmitz et al., 1999; Poritz et al., 2007; Poritz et al., 2011), which were largely associated with decreased key TJ proteins including ZO-1 and occludin. Colonic inflammation mice model generated using the dextran sulfate sodium (DSS) showed decreased ZO-1 along with consecutive increase in claudin-1 expression (Poritz et al., 2007). Similar increase in claudin-1 expression was observed in IEC-18 cells when exposed to TNF-α and in the patient samples of UC (Poritz et al., 2011). Our study on HT29 cells also showed that TNF-α regulates claudin-1 expression and localization via activation of ERK1/2 and Src signaling (Bhat et al., 2016). IL6, one of the major proinflammatory cytokine mainly produced by epithelial cells and immune cells of the lamina propria has been shown to induce claudin-2 expression through MEK/ERK, PI3K signaling pathways, and transcriptional factor Cdx2 expression (Suzuki et al., 2011). The dynamic nature, composite signaling environment and the sensitive balance between proliferation and cell shedding of the intestinal epithelium provides great potential of disturbances and an interesting area of research. This whole set of proliferation and physiologic epithelial cell shedding involves rearrangement of TJ proteins to extrude the cell from the epithelium (Martini et al., 2017). The integrity of TJs is firmly regulated by TJ proteins and Myosin light chain kinase (MLCK), an important regulatory element, is found to be deregulated in the intestine of IBD patients (Blair et al., 2006). Phosphorylation of MLCK results in F-actin reorganization and consequently TJ protein redistribution to intracellular compartments form the apical domain of the enterocyte (Shen et al., 2006) and ZO-1 exchange was suggested to be critical for this process (Yu et al., 2010). In addition, MLCK activation results in increased claudin-2 expression by stimulating IL-13 synthesis (Weber et al., 2010). In Caco2 cells, MLCK gene expression is stimulated by TNF-*α* and interleukin-1*β via* NF*κ*B resulting in enhanced TJ permeability (Ye and Ma, 2008; Al-Sadi et al., 2010). Therefore, inhibiting the TNF-α induced MLCK expression can restore the function of TJ barrier. Mice with experimental colitis had increased expression of MLCK, resulting dysregulation of TJs and a severe loss of epithelial barrier function (Su et al., 2013). Studies have also shown that MLCK-induced caveolin-1-dependent endocytosis of occludin is important for regulation of TJ structure and function (Marchiando et al., 2010b). In contrast, the TJ redistribution induced by IFN-*γ* was found to be *via* Rho/ROCK signaling-dependent macropinocytosis-like mechanism (Bruewer et al., 2005). Rho-A is also vital to epithelial integrity and Rho-A signaling has been shown to be impaired in IBD patients because of the reduced expression of the Rho-A prenylation enzyme geranylgeranyltransferase-I (Lopez-Posadas et al., 2016). Mice lacking either Rho-A or geranylgeranyltransferase-I in IECs suffered from chronic intestinal inflammation, cytoskeleton rearrangement, and aberrant cell shedding. Another important molecule involved in regulated cell shedding and epithelial integrity is Rho associated kinase, which is a downstream effector of Rho-A and plays vital role in signal transduction pathways that control adhesion, transmigration, phagocytosis, and proliferation (Benoit et al., 2009; Zihni et al., 2014; Kumper et al., 2016). Rho-associated kinase was found to be highly activated in the inflamed intestinal mucosa of patients with CD, suggesting impaired cytoskeletal rearrangements (Segain et al., 2003).

In summary, the regulated tissue specific expression of TJ proteins and their crosstalk with signaling pathways both at membrane and within the cell determines distinct functions of the small and large segments of the healthy intestine. In IBD, TJ proteins change in expression and localization which causes segment-specific alterations in paracellular barrier and channel functions. These changes generally result in increased paracellular transport of solutes and water, typically mediated by up-regulated claudin-2 and down-regulated barrier forming claudins. This whole process leads to diffusion of ions and water from blood to lumen, causing leak-flux diarrhea. The other possibility is the increased permeability to large molecules including luminal pathogens which may initiate an immune response and cause inflammation. The significant contribution of claudins in different inflammatory processes and diseases and in the recruitment of signaling molecules brands them appropriate for therapeutic intervention.

## Bridging Inflammation and Inflammation Associated Colorectal Cancer

Inflammation and cancer are closely connected. Inflammation can contribute from initiation of the malignant phenotype to metastatic spread in different ways but usually requires a switch from acute to chronic inflammation (Coussens and Werb, 2002; Raposo et al., 2015). Inflammatory cells generate reactive oxygen species and proinflammatory mediators which may enhance the mutation rate of cells, induce DNA damage and increase genomic instability (Waris and Ahsan, 2006). These reactive species may also inactivate mismatch repair functions, supporting tumor initiation. In a positive feedback loop, DNA damage can also lead to inflammation, supporting tumor progression (Ohnishi et al., 2013). Inflammation surges the risk of developing many types of cancer (including bladder, cervical, gastric, intestinal, oesophageal, ovarian, prostate and thyroid cancer) but here we will briefly review IBD associated CRC as this falls within the scope of manuscript. It is well known that patients with IBD are at higher risk of CRC. Many evidences suggest a link between inflammation and CRC (Rizzo et al., 2011; Romano et al., 2016). There is a growing evidence that supports the role of immune cells, inflammatory cells, chemokines, cytokines and proinflammatory mediators in the pathogenesis of IBD associated CRC (Mariani et al., 2014; Luo and Zhang, 2017). Inflammatory cells and mediators support the cancer growth and progression by different means which includes (1) production of ROS and RNI, both are mutagenic; (2) by supporting neo-angiogenesis, and (3) by supporting metastatic spread through the induction of EMT (Grivennikov et al., 2010; Gupta et al., 2012). The proinflammatory pathways that are involved in these processes and provide a mechanistic link between inflammation and cancer include but not limited to, NF-*κ*B, TNF-α, IL-6/STAT3, cyclooxygenase-2 (COX-2)/PGE_2_, and IL-23/Th17 (Crusz and Balkwill, 2015; Raposo et al., 2015). The above literature besides providing a link between cancer and inflammation, also suggests the importance of the epithelial cell junctions in maintaining the integrity of the intestinal epithelium. Inflammatory cytokine mediated or any other disruption to the epithelial cell junctions results in chronic intestinal inflammation predisposing to the development of tumors.

The detailed knowledge and understanding of the mechanisms that associate IBD with CRC may provide concrete benefits both in the scientific and clinical facets related to the introduction of innovative diagnostic and therapeutic measures in patients with chronic inflammations.

## Conclusion

Tight junctions have emerged as dynamic bidirectional signaling hubs which host diverse regulatory mechanisms for appropriate junction assembly and function. TJ proteins signal to the cell interior either directly or through recruiting other signaling molecules to regulate cell proliferation, migration, survival and differentiation. Several cancers and inflammatory disorders have altered expression of TJ proteins especially claudin family members, making them attractive diagnostic and prognostic markers. The functional importance of claudins in cancer progression and other inflammatory diseases is well recognized, however, the mechanisms that drive these disease processes remain poorly understood. We are still in the preliminary phase to understand the interaction between junctional membrane proteins and these signaling mechanisms. We are gradually learning how this interaction affects junctional functions on one hand, and how, on the other hand, the junctional adhesion proteins use these mechanisms to signal to the cell interior. Most of these mechanisms have been studied in isolation and, therefore, it is not clear how distinct signaling mechanisms cooperate and influence one another, and how they are triggered in response to diverse stimuli. To understand these processes is of significant biological importance in terms of pathological relevance as junction assembly is disturbed in many common diseases, including acute and chronic inflammations and different types of cancer. The large number of pathogenic viruses and bacteria that interact with TJ components are thus of great interest, as they provide excellent experimental tools to expound how the deregulation of junctional signaling mechanisms contributes to disease development. Therefore, more studies are warranted in this direction and thus the development of claudin-targeted therapeutics represents a promising endeavor.

## Author Contributions

AB and SrU: prepared the scientific material, wrote the main text, generated tables, and made figures. IA: figures revised and edited the text. SH, SY, MS, and HA-N: critical revision of the scientific contents. MH and SU: developed the structure of the review, revised the scientific material, and edited the contents. All authors read and approved the final manuscript.

## Conflict of Interest Statement

The authors declare that the research was conducted in the absence of any commercial or financial relationships that could be construed as a potential conflict of interest.
